# Bioorthogonal Click Chemistry for Antibody-Free Profiling of Acetylation, Propionylation, and Butyrylation in *Pseudomonas aeruginosa* and Methicillin-Resistant *Staphylococcus aureus*

**DOI:** 10.1021/acsinfecdis.5c00985

**Published:** 2026-02-16

**Authors:** Haley N. Monacchio, Ritika S. Shah, Christian F. Montes, Grace Z. Wang, Justin W. Walley, Chelsey M. VanDrisse

**Affiliations:** † 1355 University of Georgia, Department of Microbiology, 140 Cedar Street, Athens, Georgia 30602, United States; ‡ University of Georgia, Department of Genetics, 120 E Green Street, Athens, Georgia 30602, United States; § 1177 Iowa State University, Plant Pathology, Entomology and Microbiology, 2213 Pammel Drive, Ames, Iowa 50011, United States; ∥ 6469 California Institute of Technology, Division of Chemistry and Chemical Engineering, 221 Spalding Laboratory, Pasadena, California 91125, United States

**Keywords:** bacterial acylation, acetylation, propionylation, butyrylation, posttranslational modifications

## Abstract

Lysine acylation is a posttranslational modification (PTM) conserved in all domains of life and is essential for regulating diverse biological processes. Traditional methods for investigating acylation rely on anti-acyl-lysine antibodies, which are costly and time-consuming and often exhibit variable affinity. To remedy these pitfalls, we developed an antibody-free method for bacterial acylome enrichment using bioorthogonal click chemistry coupled with tandem mass spectrometry. We applied this approach to the pathogens *Pseudomonas aeruginosa* and methicillin-resistant *Staphylococcus aureus* (MRSA) to explore the biological significance of acylation in each organism. We characterized the acetylome, propionylome, and butyrylome in *P. aeruginosa* UCBPP-PA14 and the acetylome and propionylome in MRSA. Comparative analyses revealed unique PTM dynamics showing that acylation regulated a wide range of cellular functions, including metabolism, antibiotic resistance, virulence, and stress response. This work establishes the first antibody-free enrichment method for defining bacterial acylomes and provides new insight into global lysine acylation networks in pathogenic bacteria.

## Graphical abstract

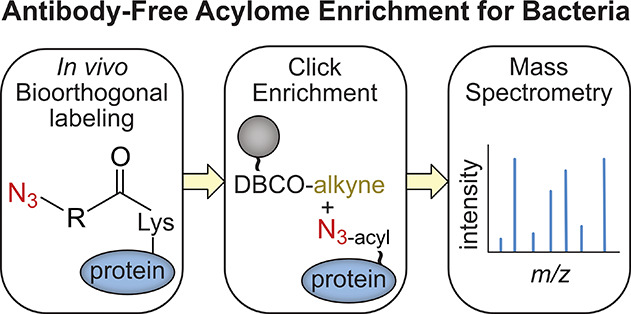

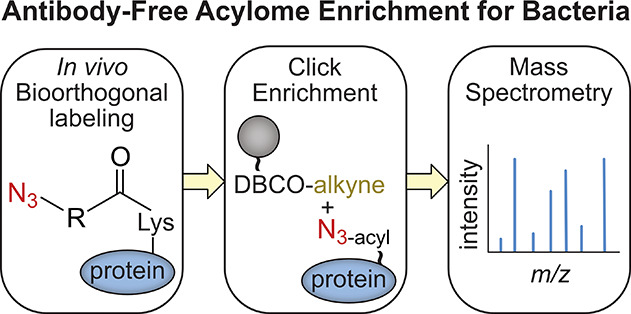

## Introduction

Posttranslational modifications (PTMs) of proteins are critical for the regulation of biological processes such as cell signaling, protein activity, protein stability, protein localization, gene expression, and cell cycle regulation.^1–3^ In bacterial pathogens, posttranslational modifications like acylation are emerging as key regulators of stress responses,^4^ virulence factor expression,^5–8^ and host–pathogen interactions.^9–11^ Despite their importance, the mechanistic roles of PTMs remain largely uncharacterized, particularly in clinically relevant organisms such as *Staphylococcus aureus* and *Pseudomonas aeruginosa*, which are major causes of hospital- and community-acquired infections. Both *P. aeruginosa* and *S. aureus* display significant antibiotic resistance and tolerance in the clinic,^12,13^ and PTMs represent an underexplored layer of regulation that could inform novel therapeutic strategies or provide insight into mechanisms that enable pathogen survival in hosts.

One particular type of PTM, acylation, is carried out by acyltransferases and controls many biological processes including transcription and metabolism in eukaryotes,^14,15^ but it is a new area of research in bacteria.^16^ Acyltransferases catalyze the transfer of an acyl group from acyl-CoA to either *N*_α_ termini of proteins or the *N*_ε_ groups of lysyl residues.^17^ While the former is irreversible,^18^ *N*_ε_ acylation is reversible and plays important roles in the regulation of bacterial transcription,^6^ translation,^19^ metabolism,^20^ and virulence.^21^ Given the large number of acyltransferases in a single bacterial genome (up to 75), identifying physiological targets of most bacterial acyltransferases is cumbersome, limiting our understanding of the regulatory networks controlled by acylation. Consequently, researchers have focused on defining the entire acylated proteome, or acylome, as a strategy to map cellular regulation by acylation in a variety of bacterial species.^22–30^

In *P. aeruginosa*, several global acylome studies have mapped the scope of lysine acylation, initially identifying a broad diversity of acetylated proteins across core metabolic, stress response, and virulence-associated pathways.^31^ Subsequent analyses profiled both acetylation and succinylation, further highlighting extensive modification of metabolic enzymes and membrane-associated proteins.^32^ More recently, a comprehensive acetylome study revealed substantial acetylation diversity across *P. aeruginosa* strains and growth conditions, underscoring the dynamic and context-dependent nature of this PTM.^29^ Similarly, in methicillin-sensitive *Staphylococcus aureus*, proteome-wide profiling of lysine acetylation and succinylation has documented widespread modification of metabolic and virulence-related proteins, implicating acylation as an important regulatory layer in this organism as well.^27,33^

Notably, these prior acetylome studies in *P. aeruginosa* and *S. aureus* primarily used anti-acyl-lysine antibody enrichment, which, while powerful, is subject to epitope bias and may not capture the full dynamic range of acylation modifications. The use of anti-acyl-lysine antibodies can cause cross-reactivity, and differential binding to acylated lysine residues.^34–36^ Furthermore, these antibodies have lower binding affinity and accessibility to the epitope on tightly folded proteins, which hamper the detection of acylated lysine residues.^37^ Lastly, probing for acyl modifications of varying chain lengths (*e*.*g.,* acetylation, propionylation, or succinylation) requires distinct antibodies for each type of modification, making comprehensive analysis challenging and resource-intensive. These limitations make it difficult to capture the full scope and diversity of acylation events, especially those occurring dynamically in response to stress or metabolic adaptation.

To overcome these pitfalls, we developed a protocol that uses bioorthogonal chemistry combined with alkyne–azide cycloaddition click chemistry^38^ to selectively label and enrich specific posttranslational modifications in the opportunistic pathogens, *P. aeruginosa* and methicillin-resistant *S. aureus*. Bioorthogonal chemistry relies on the incorporation of biomolecules with small, chemically reactive groups, such as azides, that can selectively react with probes downstream without interfering with native cellular processes.^38^ While similar protocols exist for analyzing PTMs with bioorthogonal chemistry in eukaryotes,^39–47^ to our knowledge, this is the first application of bioorthogonal click chemistry to define the acylome in any bacterial species. This technique is adaptable to different acyl analogs, offering a broadly applicable tool for dissecting PTM regulation in diverse microbial contexts. With this protocol, a purification tag or a fluorophore can then be covalently attached onto azido-acylated proteins, which allows for selective enrichment of these proteins, followed by comprehensive analysis via mass spectrometry or visualization via in-gel fluorescence (Figure 1). Labeling of acylated proteins occurs *in vivo* during a 30-to-60 min period, allowing for a dynamic snapshot of acylation events that can be analyzed with bacterial mutants or various metabolic growth states. Our method is fast, enriches for acylated proteins of interest, and enhances the signal-to-noise ratio for mass spectrometry-based proteomic analysis.

**Figure 1:**
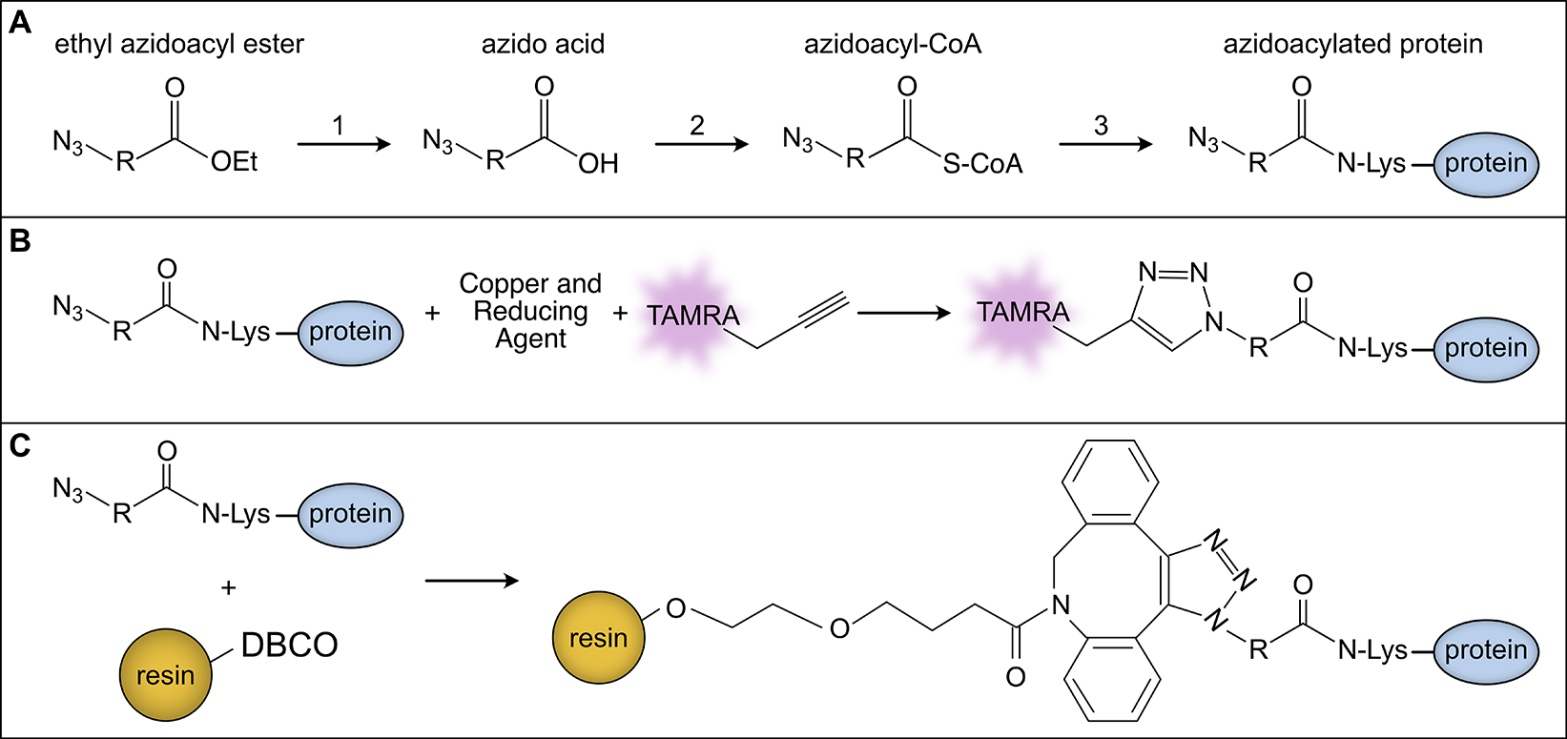
Bioorthogonal metabolic labeling and click chemistry strategies for detection and enrichment of azidoacylated proteins. (A) *In vivo* bioorthogonal incorporation of azidoacyl groups into proteins. Ethyl azidoacyl esters are hydrolyzed *in vivo* by nonspecific bacterial esterases to yield the corresponding azido acids (1). Native acyl-CoA synthetases convert azido acids to their acyl-CoA thioesters (2), which acyltransferases use to acetylate proteins (3). (B) Copper-catalyzed click chemistry (CuAAC) with alkyne–TAMRA labeling. Following cell lysis, azidoacylated proteins generated as shown in (A) are reacted with the alkyne-functionalized TAMRA fluorophore via CuAAC. Labeled proteins are separated by SDS-PAGE and detected by in-gel fluorescence imaging. (C) Copper-free click chemistry with DBCO–resin affinity enrichment. Following cell lysis, azidoacylated proteins from (A) are subjected to strain-promoted azide–alkyne cycloaddition (SPAAC) by incubation with DBCO-functionalized agarose resin. The reaction covalently links the azidoacylated proteins to the resin, enabling their affinity enrichment and subsequent downstream analysis via mass spectrometry.

In this study, we defined the acylomes for acetylation and propionylation in *P. aeruginosa* and methicillin-resistant *S. aureus* (MRSA), and butyrylation in *P. aeruginosa*. Enrichment analysis of the acylated proteins allowed for the identification of multifaceted biological processes related to metabolism, stress responses, and pathogenesis mechanisms. By providing a robust, rapid, and broadly applicable method for profiling multiple acyl modifications, this approach addresses key limitations of antibody-based strategies and opens new avenues for investigating dynamic PTMs in bacterial pathogens. Moreover, our technique has potential applications beyond the bacterial species in this study, offering a generalizable platform to explore the role of acylation in bacterial physiology and pathogenicity. By integrating chemical biology with proteomics, our approach enhances the ability to study bacterial PTMs with high specificity and temporal resolution, ultimately advancing our understanding of fundamental aspects related to the bacterial physiology of important pathogens.

## Materials and Methods

### Strains, Growth Conditions, and Chemicals

Both *Pseudomonas aeruginosa* UCBPP-PA14 and methicillin-resistant *Staphylococcus aureus* JE2 strains were grown overnight in LB broth at 37 °C with shaking at 250 rpm. *P. aeruginosa* was grown into phosphate media (adapted from ref 48) containing succinate (40 mM). *S. aureus* was grown in Complete Defined Medium (CDM) with glucose (13.88 mM) (as previously described in ref 49).

The following chemicals and buffers were used in this study: 2-azidoethanoic acid (azidoacetic acid) (Avantor VWR; TCA3079), 3-azidopropionic acid (azidopropionic acid) (Fisher Scientific; NC0906249), 4-azidobutyric acid (azidobutyric acid) (BroadPharm; BP-23875), ethyl 2-azidoethanoate (ethyl azidoacetate) (Fisher Scientific; E12555G), ethyl 3-azidopropanoate (ethyl azidopropionate) (BroadPharm; BP-29695), ethyl 4-azidobutyrate (ethyl azidobutyrate) (BroadPharm; BP-29696), BugBuster HT Protein Extraction Reagent (Sigma-Aldrich; 70922-3), 2-[4-(2-hydroxyethyl)­piperazin-1-yl]­ethanesulfonic acid (HEPES) (GoldBio; H-400–2), Copper Sulfate (Sigma-Aldrich; 20919), Aminoguanidine Hydrochloride (Fisher Scientific; AC368910250), Sodium Ascorbate (Sigma-Aldrich; 396494), tris-hydroxypropyltriazolylmethylamine (THPTA) (Vector Laboratories; CCT-1010-100), and tetramethylrhodamine (TAMRA) alkyne dye (Click Chemistry Tools; 1255-5). Lysostaphin (Fisher Scientific; NC0199495), SDS Micropellets (Fisher Scientific; BP8200500), Chloroacetamide (VWR; TCC2536-5G), Urea (Fisher Scientific; U15-3), Sodium Chloride (Fisher Scientific; BP35810), DBCO-agarose beads (Vector Laboratories; CCT-1034), DTT (GoldBio; DTT10), Tris Base (Fisher Scientific; BP1525), Ammonium Bicarbonate (VWR; 101202-992), and Sequencing Grade Trypsin (Promega Corporation; V5111).

### Growth Curves

Overnight cultures were diluted to an OD_600_ of 0.4 in the respective minimal medium and incubated with varying concentrations of azidoacids or ethylazidoacids in a 96-well plate at 37 °C (gradient of 1 °C over the height of the plate to prevent condensation) with constant shaking. OD_500_ measurements were taken every 30 min over a period of 24 h (49 reads in total) using the Agilent BioTek Epoch 2 Microplate Spectrophotometer (with continuous orbital shaking [4 mm]; read speed: normal; delay: 100 ms). Each condition contained biological triplicates with error bars representing the standard deviation.

### Azido-Functionalized Compound Treatment and Sample Collection

Briefly, overnight cultures were subcultured into minimal medium with 40 mM succinate in a 250 mL flask at a starting OD_600_ of 0.08 and incubated at 37 °C with shaking at 250 rpm until the OD_600_ was 0.4–0.5. Once the desired OD_600_ was achieved, the culture was split into 18 mm glass tubes (5 mL liquid in each) and treated with 10 mM (for *P. aeruginosa*) or 25 mM (for *S. aureus*) of each of the following chemicals: azidoacetic acid, azidopropionic acid, azidobutyric acid, ethyl azidoacetate, ethyl azidopropionate, and ethyl azidobutyrate (a negative control, where medium with no chemical was also maintained). The chemicals were diluted (200 mM for PA and 500 mM for SA) in the medium used for growth such that 250 μL of each diluted chemical was added to 5 mL of the culture to achieve the desired concentration (10 mM for *P. aeruginosa* and 25 mM for *S. aureus*). These were then reincubated at 37 °C with shaking at 250 rpm for 30 min, following which they were pelleted and stored at −80 °C.

### Fluorescent Labeling of Azide-Modified Proteins via Copper-Catalyzed Azide–Alkyne Cycloaddition

For *P. aeruginosa*, cell pellets were thawed at room temperature and lysed with BugBuster HT Protein Extraction Reagent (100 μL) for 20 min on a rocking platform. *S. aureus* cells were lysed in BugBuster HT Protein Extraction Reagent (100 μL) supplemented with lysostaphin (100 μg mL^–1^). Lysates were clarified by centrifuging at 21,300 × *g* for 10 min at room temperature. The supernatant was pipetted into a fresh tube, and the protein concentrations were measured using a Bradford Assay with a standard curve (BioRad). Each click chemistry reaction (250 μL) contained protein (75–100 μg), HEPES (10 mM, pH 7.5), CuSO_4_ (100 μM), THPTA (500 μM), TAMRA–alkyne (2.5 μM), Aminoguanidine HCl (45 mM), Sodium Ascorbate (45 mM), and water. CuSO_4_, THPTA, and TAMRA–alkyne were premixed and incubated in the dark for a minimum of 3 min and added to the protein mixture with HEPES. The reaction was initiated by the addition of the aminoguanidine hydrochloride and sodium ascorbate, followed by gentle mixing via tube inversion. The reaction proceeded in the dark for 30 min at room temperature. Protein was extracted by the addition of 400 μL methanol, 100 μL chloroform, and 300 μL water (samples were vortexed after the addition of each chemical). The samples were spun down at 21,300 × *g* for 3 min. The top layer was removed (care was taken to not disturb the wafer-thin protein layer at the boundary of the two layers), following which the samples were washed twice with 400 μL of methanol by centrifuging at 21,300 × *g* for 3 min and the supernatant was discarded. The tubes were left open to allow evaporation of any residual methanol. Protein pellets were suspended in 25 μL of a 1:1 mix of 4× Laemmli Sample Buffer and BugBuster HT Protein Extraction Reagent, followed by heating at 95 °C for 5 min. 20 μL of each sample was then loaded onto 4–20% Mini-PROTEAN TGX Precast Protein Gels (purchased from BioRad; 4561093); the gels were run at 200 V for 45 min. Using a Typhoon scanner, the gels were imaged for TAMRA fluorescence. The same gel was then stained with Coomassie Blue and Fairbanks Destaining Solution and destained with water. The destained gels were then imaged.

### Preparation of Azidoacyl-Labeled Samples for Mass Spectrometry

The same growth conditions and azido treatments were followed as explained above except overnight cultures were subcultured into 100 mL of the appropriate minimal medium and carbon source and diluted to a starting OD_600_ of 0.1. Once an OD_600_ between 0.4 and 0.5 was achieved, azido compounds were diluted to a working stock in the coordinating minimal medium (50 mM). *P. aeruginosa* cultures were then treated with ethyl azidoacetate (5 mM), azidopropionic acid (5 mM), azidobutyric acid (5 mM), or dimethyl sulfoxide (DMSO). DMSO-treated samples were used as a negative control to account for nonspecific binding to the DBCO-agarose beads during enrichment. *S. aureus* cultures were treated with either ethyl azidoacetate (5 mM), azidopropionic acid (5 mM), or DMSO. For each compound and organism, cultures were grown in biological quintuplicate.

### Protein Extraction and Click Chemistry Enrichment for Proteomic Analysis

For *P. aeruginosa*, cell pellets were thawed at room temperature and lysed with BugBuster HT Protein Extraction Reagent (500 μL) supplemented with 100× Halt Protease Inhibitor Cocktail (5 μL) for 20 min on a SCILOGEX SCI-0180-S orbital shaker at 70 rpm. For *S. aureus*, cell pellets were lysed with BugBuster HT Protein Extraction Reagent (500 μL), lysostaphin (100 μg mL^–1^), and 100× Halt Protease Inhibitor Cocktail (5 μL) for 30 min at 37 °C shaking at 70 rpm. The lysates were spun at 21,300 × *g* for 10 min at room temperature. Supernatants were removed and placed into a fresh 1.7 mL tube, and protein concentrations were measured by Bradford Assay (BioRad) using a BioTek Epoch 2 Microplate Spectrophotometer in a 96-well plate format with pathcheck correction. A final concentration of 3 mg mL^–1^ of each lysate was achieved by diluting the samples up to 1 mL with SDS (1% w/v in PBS). Freshly made chloroacetamide (120 mM, suspended in SDS (0.8% w/v in PBS)) was added to the samples to block free thiol groups from forming disulfide bonds. Samples were incubated at 65 °C in the dark, shaking at 1,200 rpm for 30 min using an Eppendorf Thermomixer R. Next, to denature the proteins and improve accessibility of the azide groups for the click reaction, Urea (1.6 M) and NaCl (0.17 M) in PBS solution were added to the samples. DBCO-agarose beads, containing a cyclooctyne ring with an alkyne group, were washed three times at a 1:1 ratio with SDS (0.8% w/v in PBS) at 500 × *g* for 1 min. To allow the click reaction to occur, washed and resuspended beads (1.2% v/v) were added to the protein samples and incubated at room temp, in the dark, spinning at 70 rpm on a Thermo Scientific Digital Cel-Gro Tissue Culture Rotor overnight.

The following day, in a biological safety cabinet, the supernatant was removed and the beads were washed with water (1 mL). DTT (1 mM) in SDS (0.8% w/v in PBS) was added to each sample and incubated at 70 °C for 15 min. After incubation, the supernatant was discarded and fresh chloroacetamide (40 mM) was added to prevent the reformation of disulfide bonds. The samples were incubated at room temperature for 30 min in the dark, spinning at 70 rpm on the Thermo Scientific Rotor. Next, the resin was transferred to a BioRad Poly-Prep Chromatography Gravity Column and washed eight times with 5 mL of SDS (0.8% w/v in PBS) and Urea (8 M) in Tris (1 M, pH 8), second washes were capped and incubated at room temperature for 30 min. Lastly, the columns were washed eight times with 5 mL of acetonitrile (ACN) (20% v/v), and the columns were capped and incubated at room temperature for 10 min. After the final wash, the bottom of the columns was capped and the resin was resuspended with ACN (10% v/v in ammonium bicarbonate (50 mM)). Once resuspended, the beads were transferred to a fresh 1.7 mL epi tube and spun at room temp for 5 min at 2,000 × *g* and the supernatant was removed until 100 μL remained. To generate peptides, sequencing-grade trypsin (1 ng μL^–1^) dissolved in ACN (10% w/v in ammonium bicarbonate (50 mM)) was added to each sample. The tubes were placed in an Eppendorf Thermomixer R at 37 °C shaking at 1,200 rpm for 18 h.

The next day, in a biological safety cabinet, the samples were spun down, and the supernatant containing digested peptides was transferred to a fresh 1.7 mL tube. The resin was washed twice with 150 μL of ACN (20% v/v), and the washes were combined with the supernatant. The samples were spun for a final time, and the remaining supernatant was added to the fresh tube. The compiled supernatant tubes were placed in an Eppendorf Vacufuge Plus at 4 °C overnight until fully dry. Samples were placed at −20 °C until they were shipped for analysis by liquid chromatography-tandem mass spectrometry (LC-MS/MS). Proteins were eluted from the beads and digested into peptides using S-Trap sample processing technology (ProtiFi, Fairport, NY, USA) previously described.^50^

### LC-MS/MS Analysis of Peptide Samples

An equal volume of the recovered sample was analyzed by LC-MS/MS as follows. Chromatography was performed on a Thermo Vanquish Neo UHPLC instrument in “heated trap-and-elute, backward flush” mode. Peptides were desalted and concentrated on a PepMap Neo trap column (300 μM i.d. × 5 mm, 5 μm C18, 100 Å μ-Precolumn, Thermo Scientific) at a flow rate of 5 μL min^–1^. Sample separation was performed on an Aurora Ultimate Column (IonOpticks) with a flow rate of ∼350 nL min^–1^ over a 60 min reverse-phase gradient, followed by a column/trap wash at 80% ACN for 10 min. Eluted peptides were analyzed using a Thermo Scientific Orbitrap Exploris 480 mass spectrometer with a FAIMS pro Duo interface installed, which was directly coupled to the UHPLC through a Flex Ion source (Thermo Scientific). Data Independent Acquisition (DIA) was performed using Xcalibur 4.0 software in positive ion mode with a spray voltage of 2.0 kV, a capillary temperature of 280 °C, an RF of 45, a FAIMS compensation voltage of −45, and a total carrier gas flow of 4.2 L/min. A full scan was acquired at a resolution of 60,000, a scan range of 400–900, a normalized automatic gain control (AGC) target % of 225, an absolute AGC value of 2.25e6, and a maximum inject time set to auto. Forty-two DIA acquisition windows of 12 m/z with 1 Da overlap were measured at a resolution of 30,000 with a normalized AGC target % of 800, an absolute AGC of 8e5, and auto maximum ion time, over a precursor mass range of 400–900 m/z. A scan range of 145–1450 m/z and a normalized collision energy of 29 were used.

### Proteomics Data Analysis

Raw data were analyzed using Spectronaut version 19 (Biognosys). Spectra were searched, using the Pulsar search engine against the *P. aeruginosa* UCBPP-PA14 (Genome assembly ASM1462v1) and *Staphylococcus aureus* JE2 (Genome assembly ASM208552v1) reference genomes downloaded from NCBI. The spectra search was performed in “direct DIA” mode. Carbamidomethyl cysteine was set as a fixed modification, while methionine oxidation and protein *N*-terminal acetylation were set as variable modifications. Digestion parameters were set to “specific” and “Trypsin/P, LysC”. Up to two missed cleavages were allowed. A false discovery rate, calculated using a “KR” decoy generation rule, of less than 0.01 at the peptide spectral match, peptide, and protein group identification level was required. A protein was considered a hit if peptides were present in 2/5 quintuplets.

### Filtering of Mass Spectrometry Data and Identification of Unique PTMs

To correct for background signal in our acylation datasets, protein-level quantifications from the DMSO control were compared to those observed under each acylation condition. For each protein, the average log_2_ fold-change, along with the corresponding *p*-value and *Q*-value, was calculated by statistical comparison with the DMSO control. Only proteins with statistically significant enrichment (above the DMSO background, log_2_-fold change above 2.0, with *Q*-value < 0.05) were retained for inclusion in the final acylation data set (Figures S1–S5). Proteomics data from each experimental condition were processed to retain only protein groups with a positive fold-change with contaminant proteins removed. Protein group identifiers (NCBI) were used for comparison of acetylation, propionylation, and butyrylation data sets. To determine unique protein groups for each PTM, the processed csv files for each condition were loaded into Python (version 3.7.6) using pandas (version 0.24.2). For each file, protein group identifiers present in the test conditions but absent from the other data sets were identified as unique. The unique rows for each sample were exported to separate csv files for downstream analysis. Proteins shared between multiple or all datasets were found by taking the intersection of sets and relevant data exported.

### Published *Pseudomonas aeruginosa* UCBPP-PA14 Acetylome Comparison Analysis

A list of acetylated proteins was generated from *Pseudomonas aeruginosa* UCBPP-PA14 published data sets.^31,32^ UniProt identifiers from the published acetylome list were compared with those identified in the current study (total proteins and log_2_ fold-change dataset). UniProt identifiers for acetylated proteins in this study were assigned by matching NCBI Protein IDs to UniProt IDs using BioCyc Smart Tables.^51^ A comprehensive matrix was constructed encompassing all unique UniProt identifiers found in either the published dataset or the current study. For each protein, binary indicators (“X”) were used to represent acetylation hits in each study. Set operations (intersection, union, and difference functions) were used to quantify the overlap and uniqueness of acetylated proteins among the three datasets and grouped to detect unique and shared protein combinations.

### KEGG Enrichment of Unique Acylated Proteins from *Pseudomonas aeruginosa*

A gene-to-pathway mapping table for *P. aeruginosa* UCBPP-PA14 was downloaded from the KEGG database (strain identifier pau). Using data sets that contain the uniquely acylated protein hits, all associated KEGG pathway identifiers were assigned by mapping locus tags to pathway entries. Pathway descriptions were incorporated from the matching KEGG pathway description table. To assess pathway enrichments, unique locus tags from the sample sets were compared with the full background set of KEGG-annotated *P. aeruginosa* genes. For each KEGG pathway, the number of sample and background genes present or absent in the pathway was tabulated. Fisher’s exact test was used to evaluate the statistical significance of pathway enrichment among the sample genes, and the results were ranked by −log_10_-transformed *p*-values. All analyses were performed in Python (version 3.7.6) using pandas and scipy.stats.

### KEGG Enrichment of Unique Acylated Proteins from *Staphylococcus aureus*

There is no set of pathway descriptions for genes in *Staphylococcus aureus* JE2. To enable KEGG pathway annotation for *S. aureus* JE2, locus tags and protein accessions were mapped between the JE2 strain and the reference *S. aureus* USA300 (TCH1516) proteome. Protein sequence similarity between *S. aureus* JE2 and USA300 TCH1516 was assessed by all-versus-all BLASTP analysis. Of the 2,720 JE2 proteins, 2,715 (99.82%) had a corresponding ortholog in TCH1516 with at least 80% amino acid identity and 80% coverage. GenBank and GFF annotation files for both strains were downloaded, and custom parsing scripts were used to extract protein-coding sequences (CDS) and gene features, linking JE2 proteins to USA300 locus tags. Protein sequences lacking clear mapping were aligned by BLAST against the USA300 protein database to assign locus tags where possible. Gene-to-pathway mapping tables for the USA300 strain were retrieved from KEGG (strain identifier sax). KEGG enrichment using Fisher’s exact test was carried out as described above for *Pseudomonas*.

### Comparative Analysis of the MRSA and MSSA Acetylomes

To compare the acetylated proteome identified in this study (MRSA) with the previously published acetylome of Bian et al. (MSSA),^33^ we first mapped all proteins to a common identifier space. For our dataset, NCBI RefSeq protein IDs were converted to UniProt accessions using the UniProt ID mapping service. The resulting UniProt accessions were then filtered to retain only entries corresponding to *Staphylococcus aureus* subsp. *aureus* STAA8, matching the strain background used by Bian et al. UniProt-based protein lists (MSSA and MRSA) were compared by using Python. Proteins unique to our MRSA data set were analyzed via KEGG pathway enrichment with Fisher’s exact test as described above.

## Results

### Azidoacids and Ethyl Esters Selectively Label Acylated Proteins in *Pseudomonas aeruginosa* and *Staphylococcus aureus*

We sought to compare the dynamics of acetylation, propionylation, and butyrylation between *Pseudomonas aeruginosa* UCBPP-PA14 and methicillin-resistant *Staphylococcus aureus* (MRSA) and to determine whether each organism exhibits a distinct acylome profile. While extensive acetylome analyses have been performed in *P. aeruginosa* PAO1 and UCBPP-PA14,^29,31,32^ as well as methicillin-sensitive *S. aureus* (MSSA) strains,^27^ no studies to date have characterized propionylomes or butyrylomes in any *Pseudomonas* or *Staphylococcus* species. Moreover, no acylome studies have been conducted in an MRSA strain. To address these gaps, we used azidoacetate, azidopropionate, and azidobutyrate compounds as bioorthogonal labeling probes to monitor posttranslational acylation through click chemistry-based enrichment followed by SDS-PAGE and mass spectrometry quantification in *P. aeruginosa* and MRSA (Figure S6). Previous studies in mammalian cells suggest that these azido compounds could be recognized and incorporated as PTMs by the endogenous mammalian acyltransferase machinery.^47^

To test whether these azido analogs could effectively label acylated proteins in *P. aeruginosa* and *S. aureus*, we first examined their cellular uptake and incorporation efficiency. Previous studies have shown that masking the polar carboxylate group of azido acids with ester moieties enhances membrane permeability and cellular uptake, increasing their utility as metabolic probes.^47^ Once inside the cell, endogenous esterases hydrolyze the ethyl ester to release the free azido acid, which is then converted to its CoA thioester by endogenous acyl-CoA synthetases (Figure 1). The resulting azidoacyl-CoAs serve as substrates for acyltransferases, enabling azide-functionalized acylation of lysine residues via posttranslational modification. To identify conditions that supported labeling without imposing metabolic stress, we determined the sublethal concentrations for each compound in both species. The lowest noninhibitory concentrations across all compounds were 10 mM for *P. aeruginosa* and 25 mM for *S. aureus* (Figures 2A–F and 3A–F). After treatment with azido compounds, cells were lysed and subjected to copper­(I)-catalyzed azide–alkyne cycloaddition with a TAMRA–alkyne fluorophore. Labeled proteins were then resolved by SDS-PAGE and visualized by in-gel fluorescence to assess the incorporation efficiency.

**Figure 2:**
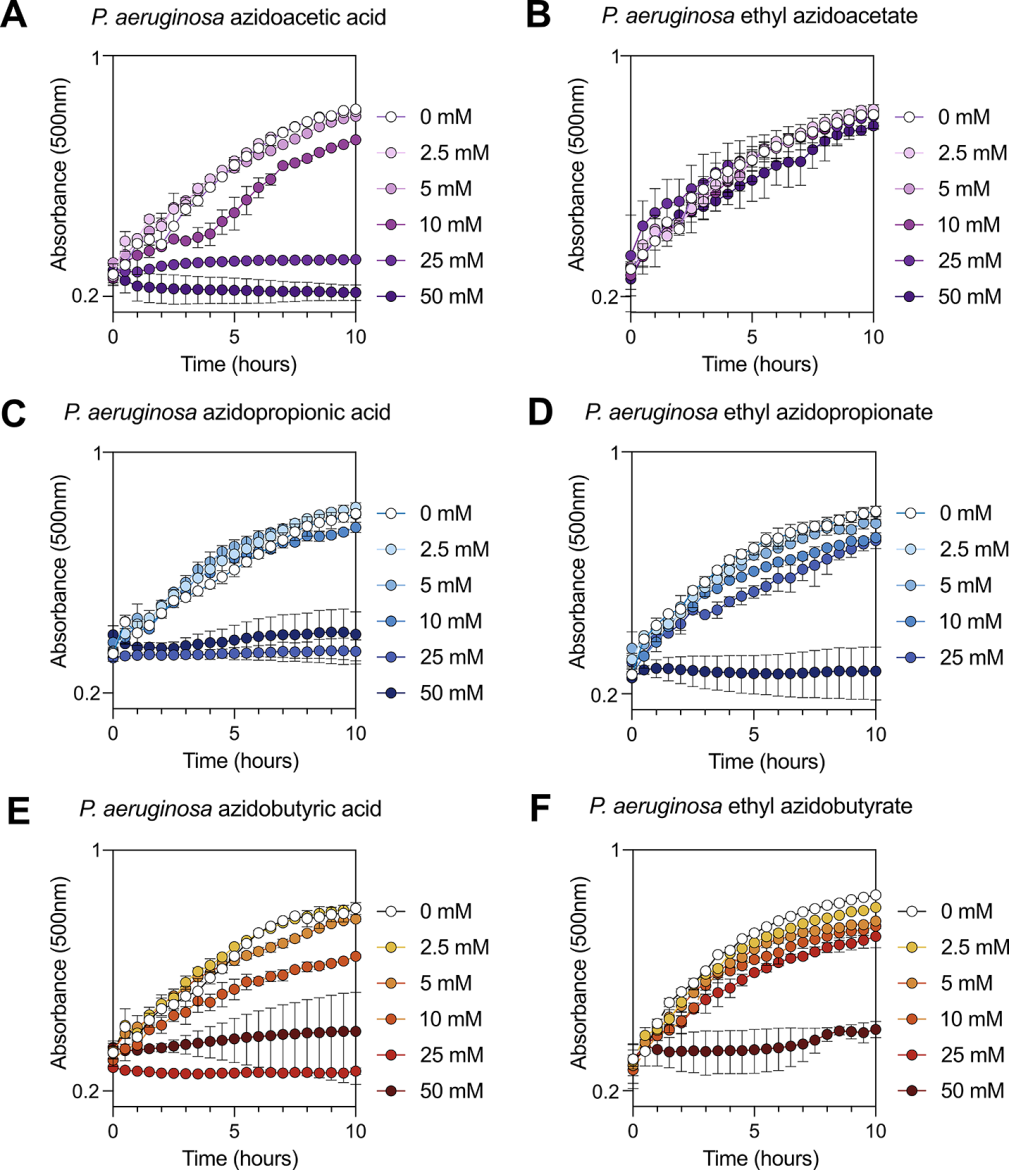
Comparative growth curves of *P. aeruginosa* upon exposure to azidoacids and ethyl azidoacyl compounds. *P. aeruginosa* was diluted to an OD_500_ of 0.4 in phosphate medium with succinate and exposed to different concentrations of (A) azidoacetic acid, (B) ethyl azidoacetate, (C) azidopropionic acid, (D) ethyl azidopropionate, (E) azidobutyric acid, and (F) ethyl azidobutyrate. The experiment was performed in a 96-well microplate, and OD_500_ readings were taken every 30 min for a period of 24 h using the Agilent BioTek Epoch 2 Microplate Spectrophotometer (with continuous orbital shaking [4 mm]; read speed: normal; delay: 100 ms). Symbols adjacent to graphs display concentrations and their coordinating colors.

**Figure 3:**
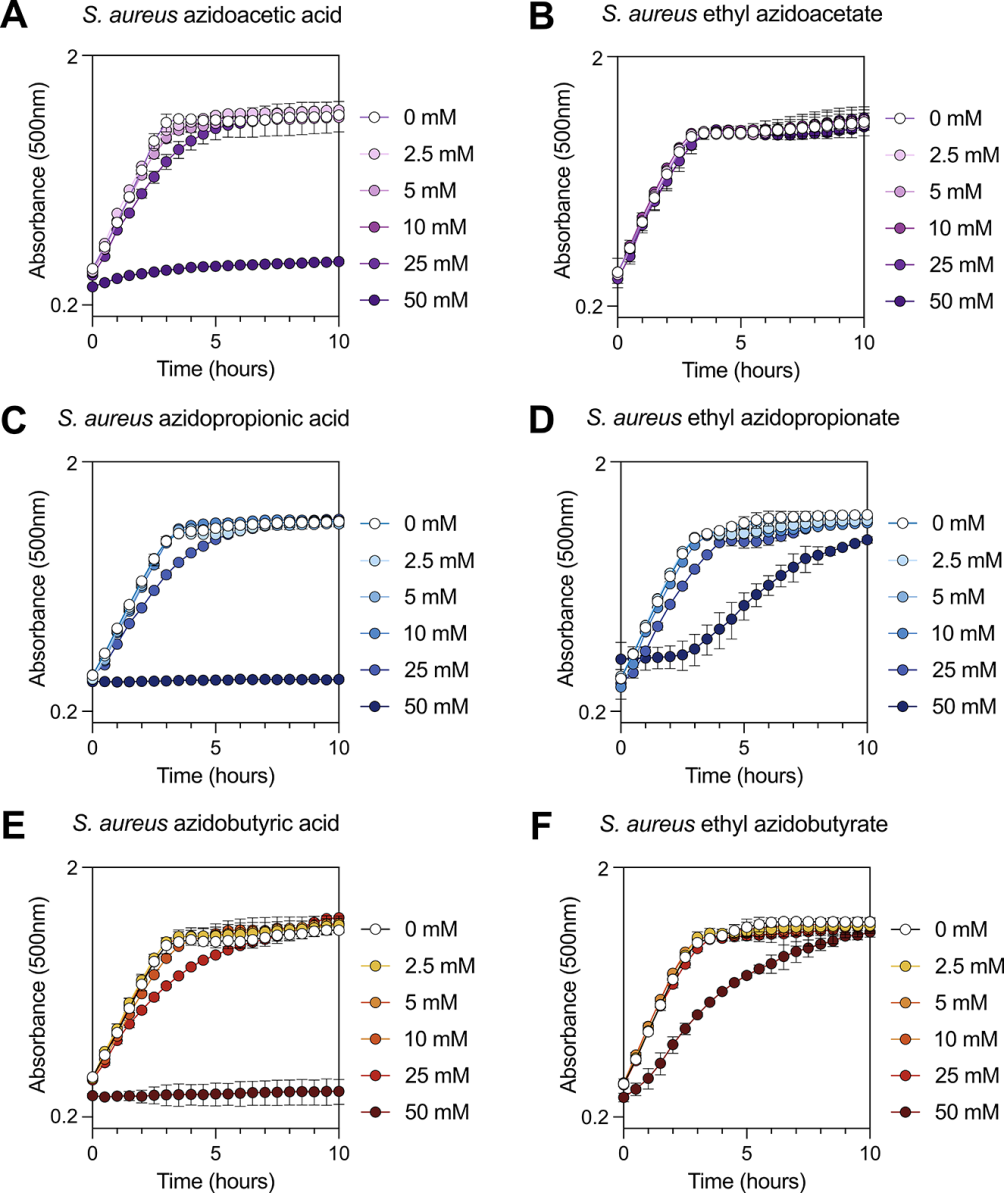
Comparative growth curves of *S. aureus* upon exposure to azidoacids and ethyl azidoacyl compounds. *S. aureus* was diluted to an OD_500_ of 0.4 in complete defined medium with glucose (13.88 mM) and exposed to different concentrations of (A) azidoacetic acid, (B) ethyl azidoacetate, (C) azidopropionic acid, (D) ethyl azidopropionate, (E) azidobutyric acid, and (F) ethyl azidobutyrate. This experiment was performed in a 96-well microplate, and OD_500_ readings were taken every 30 min for a period of 24 h using the Agilent BioTek Epoch 2 Microplate Spectrophotometer (with continuous orbital shaking [4 mm]; read speed: normal; delay: 100 ms). Symbols adjacent to graphs display concentrations and their coordinating colors.

In *P. aeruginosa*, ethyl azidoacetate produced stronger labeling of acetylated proteins compared to its azido acid counterpart (Figure 4A). In contrast, the azido acid forms of azidopropionic acid and azidobutyric acid yielded more efficient labeling than their corresponding ethyl esters. In *S. aureus*, ethyl azidoacetate similarly showed greater efficiency for detecting acetylation, whereas azidopropionic acid was more suitable for detecting propionylation (Figure 4C). For *S. aureus*, neither azidobutyric acid nor ethyl azidobutyrate produced detectable labeling above background levels (Figure 4C). Coomassie-stained SDS-PAGE gels (Figure 4B for *P. aeruginosa*, and Figure 4D for *S. aureus*) confirmed equal protein loading across all samples, indicating that observed differences in fluorescence intensity reflect labeling efficiency rather than variations in protein abundance. Identical images were analyzed to normalize brightness and contrast to the azidopropionic acid samples for improved visualization (Figure S7).

**Figure 4:**
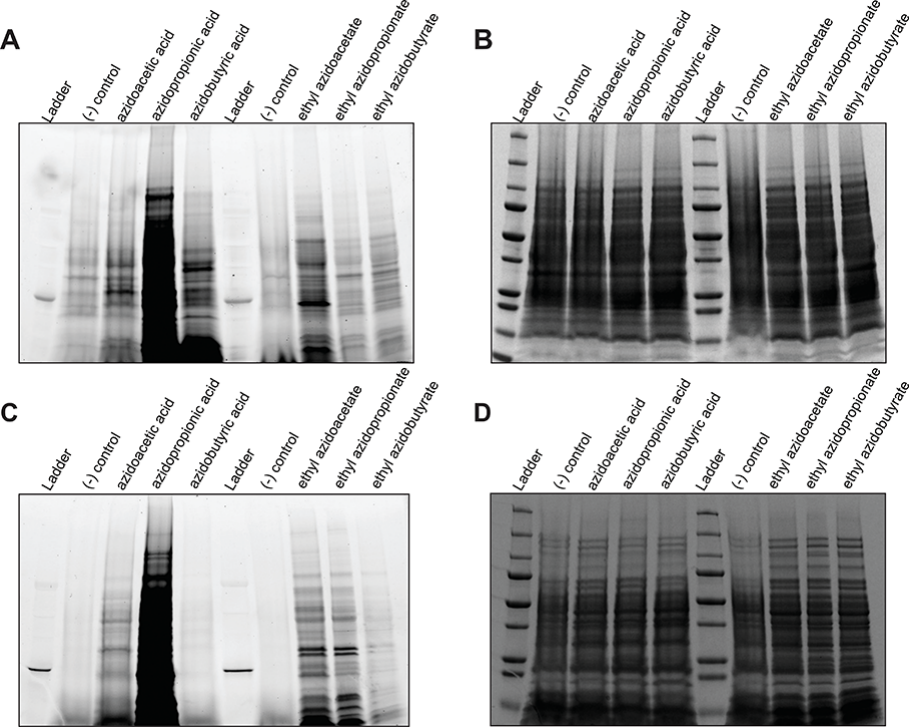
Visualization of protein acetylation, propionylation, and butyrylation in *P. aeruginosa* and *S. aureus*. *P. aeruginosa* (A and B) and *S. aureus* (C and D) were treated with azidoacids and ethyl azidoacyl esters for 30 min and samples were collected (as described in Materials and Methods). The acetylated, propionylated, and butyrylated proteins were conjugated to TAMRA alkyne fluorophore via click chemistry and the gels were visualized using the Typhoon (A and C). The same gels were then stained and destained with Coomassie Blue and Fairbanks Destaining Solution, respectively; the stained gels were then imaged (B and D). TAMRA-gels were reanalyzed to normalize to azidopropionic acid in Figure S7.

### Comprehensive Acylome Profiling Reveals the Uniqueness and Overlap of Lysine Modifications in *P. aeruginosa*

Based on the observed fluorescent labeling patterns, ethyl azidoacetate, azidopropionic acid, and azidobutyric acid were selected for subsequent acylome analyses for *P. aeruginosa*. Using strain-promoted azide–alkyne cycloaddition (SPAAC)-based enrichment combined with LC-MS/MS, we profiled the acetylated, propionylated, and butyrylated proteomes (*i.e.*, the acetylome, propionylome, and butyrylome) of *P. aeruginosa*. Applying a log_2_ fold-change cutoff relative to the negative control (as described in Materials and Methods), we identified 1,147 acetylated, 92 propionylated, and 531 butyrylated proteins (Tables S1–S3), from the 6021 proteins in the proteome.^51^ To assess the overlap among these PTMs, we compared the enriched protein sets across the three acylation modifications. This analysis revealed 712 proteins unique to acetylation, 11 unique to propionylation, and 108 unique to butyrylation (Figure 5A, Tables S4–S6). Forty-seven proteins were shared among all three PTMs, 365 proteins shared between acetylation and propionylation, 11 between propionylation and butyrylation, and 23 shared between acetylation and butyrylation. Together, these data demonstrate that our approach successfully captured the acetylome, propionylome, and butyrylome of *P. aeruginosa*, highlighting the extensive nature of lysine acylation in this organism.

**Figure 5:**
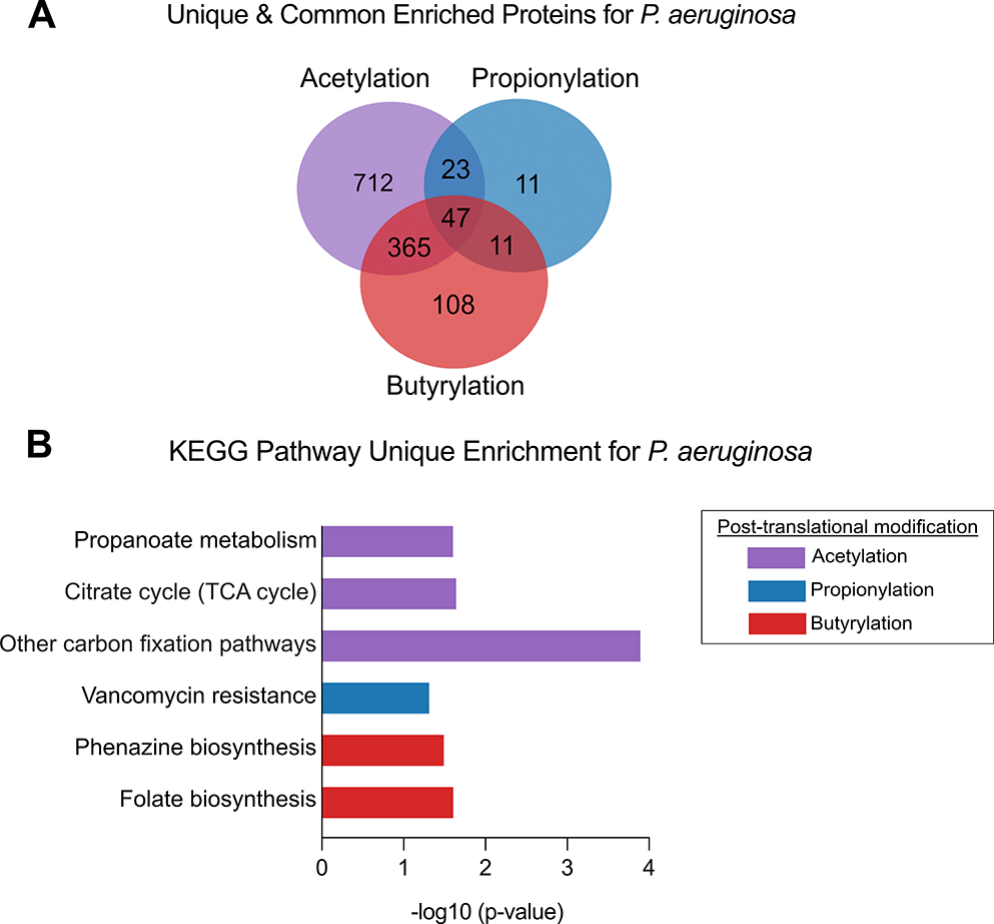
Analysis of common and unique acetylated, propionylated, and butyrylated proteins in *P. aeruginosa*. Proteins significantly enriched for each modification were identified and compared to determine unique and common acylated proteins (as described in the Materials and Methods). (A) Venn diagram showing unique and shared enriched acylated proteins. Acetylated proteins are colored purple, propionylated blue, and butyrylated in red. (B) KEGG pathway enrichment was performed on the unique enriched proteins for each modification (as described in Materials and Methods). Statistically significant KEGG pathways were determined as having a −log_10_-transformed *p*-value (*x*-axis) of 1.3 and above. The KEGG pathway (*y*-axis) was identified for unique enriched acetylated (purple), propionylated (blue), and butyrylated (red) proteins.

### Distinct Metabolic and Stress-Response Pathways Are Associated with Acetylation, Propionylation, and Butyrylation in *P. aeruginosa*

To better understand the processes regulated by acetylation, propionylation, and butyrylation in *P. aeruginosa*, a KEGG pathway enrichment analysis was performed as described in the Materials and Methods. For *P. aeruginosa*, 46% of acetylated, 45% of propionylated, and 41% of butyrylated proteins were mapped to KEGG pathways. This level of coverage demonstrates that our method effectively identifies proteins involved in known biological pathways while also enriching for hypothetical or poorly annotated proteins that are not yet represented in KEGG. Acetylated *P. aeruginosa* proteins were enriched in carbon fixation pathways, the TCA cycle, and propanoate metabolism (Figure 5B, Table S7), consistent with observations in other bacteria.^52^ Propionylated proteins were enriched in pathways associated with cell wall remodeling and stress response including those annotated under vancomycin resistance (Figure 5B, Table S8). However, *P. aeruginosa* exhibits intrinsic resistance to vancomycin, primarily due to its low outer membrane permeability, which limits the entry of large glycopeptide antibiotics.^53,54^ Due to this, *P. aeruginosa* as well as most Gram-negative pathogens does not carry vancomycin resistance genes. Based on these findings, we hypothesize that this enriched propionylated protein, a putative alanine racemase, contributes to cell wall synthesis and structural integrity, highlighting the need for critical interpretation of annotations lacking experimental validation. Butyrylated proteins were enriched in folate and phenazine biosynthesis pathways, suggesting their involvement in specialized secondary metabolism (Figure 5B, Table S9). Folate is an important metabolite and cofactor for the synthesis of essential metabolites and peptidoglycan^55^ whereas phenazines are redox-active pigments that contribute to electron transfer, biofilm formation, and virulence.^56^ Overall, we demonstrated that acetylation, propionylation, and butyrylation in *P. aeruginosa* are associated with distinct biological processes. Our method successfully identified statistically enriched proteins across diverse metabolic and stress-response pathways, providing a comprehensive understanding of PTM dynamics in *P. aeruginosa*.

### Comparison of the *P. aeruginosa* Acetylome to Published Datasets Validates the Chemical-Labeling Approach

We next compared our *P. aeruginosa* acetylome data to two previously published datasets that analyzed *P. aeruginosa* UCBPP-PA14 acetylomes.^31,32^ Both of these studies enriched for acetylated proteins using anti-acetyl-lysine antibodies, providing useful benchmarks for evaluating the robustness and generality of our chemical-labeling approach. Because antibody-based acetylome studies do not include an unenriched input control, quantitative fold-change values cannot be calculated. Therefore, we assessed total dataset overlap by comparing our acetylome without applying a log_2_ fold-change threshold. Our acetylated dataset captured 82% of the proteins identified by Gaviard et al. and 68% of those reported by Ouidir et al. (Tables S1 and S10). Overlapping and unique proteins between the studies were identified by comparing all proteins that were significantly enriched over log_2_ fold-change relative to our negative (DMSO) control. Across the three studies, we identified 858 acetylated proteins unique to our dataset, 199 unique to Gaviard et al., and 105 unique to Ouidir et al. (Figure 6A). We identified 159 proteins shared between our study and Gaviard et al., 44 between our study and Ouidir et al., and 85 between Gaviard et al. and Ouidir et al. Sixty-eight proteins were shared between all three acetylomes and were enriched for KEGG pathways related to central carbon metabolism, energy production, amino acid biosynthesis, and secondary metabolism (Figure 6B, Table S11). Together, these comparisons demonstrate that our method yields substantial overlap with antibody-based acetylomes while identifying a greater number of unique acetylated proteins, reflecting enhanced sensitivity and broader proteome coverage. Lastly, we also saw overlap with *N*-terminal acetylation data sets from *P. aeruginosa*, indicating our method may also be useful for identifying *N*_α_-acylation events^57^ (Table S1).

**Figure 6:**
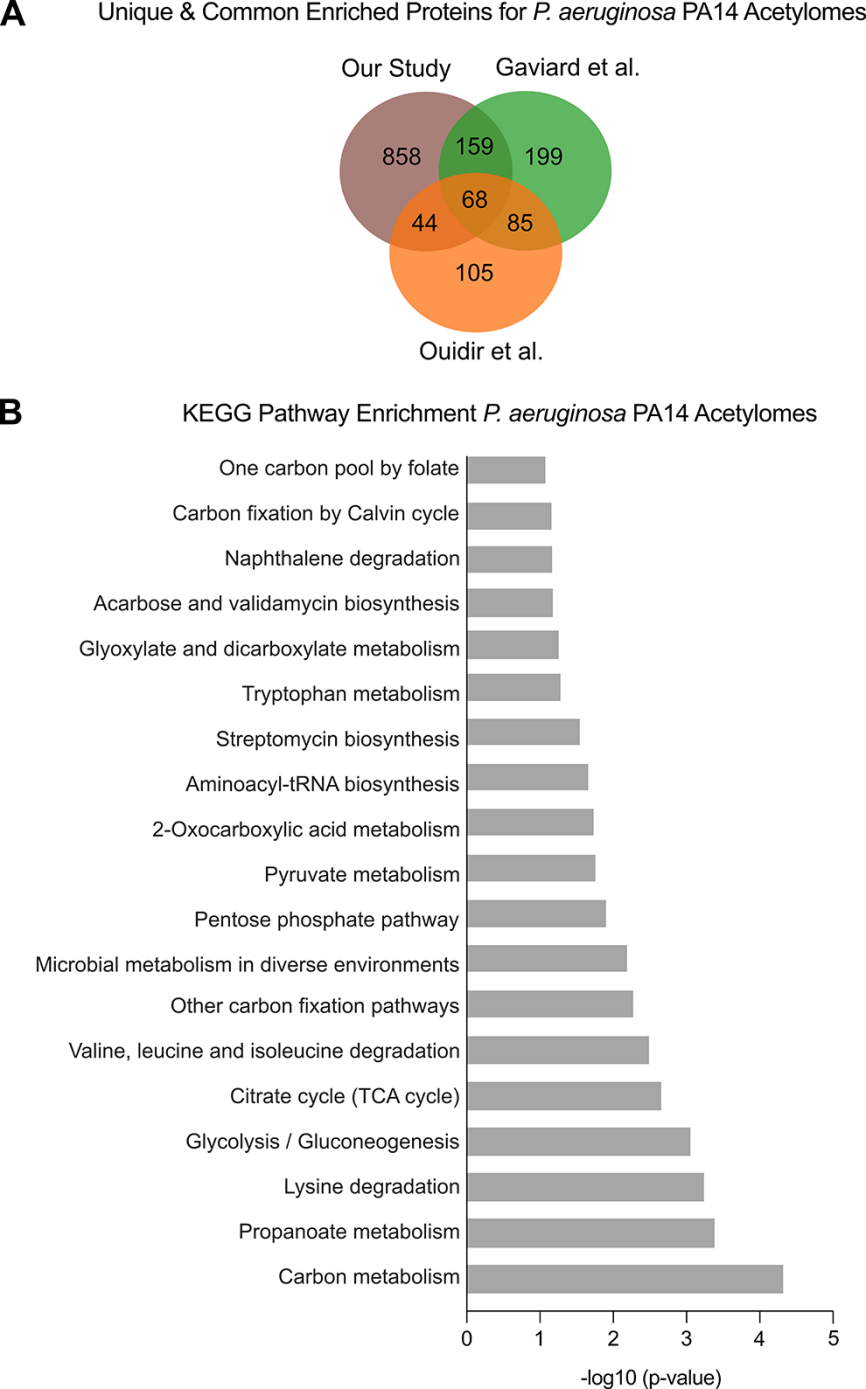
Comparison of *P. aeruginosa* acetylome to published acetylome datasets. Proteins unique and shared between our defined acetylome and two published datasets were identified as described in Materials and Methods. (A) Venn diagram showing unique and common proteins among our study (brown), Ouidir et al. (orange), and Gaviard et al. (green). (B) KEGG pathway enrichment was performed (as described in the Materials and Methods) on the 68 identified proteins shared between all three acetylomes. Statistically significant KEGG pathways were determined as having a −log_10_-transformed *p*-value (*x*-axis) of 1.3 and above.

### Acylation Mapping in *S. aureus* Reveals Distinct Regulatory Roles for Acetylation and Propionylation

Based on the observed fluorescent labeling patterns, ethyl azidoacetate and azidopropionic acid were selected for subsequent acylome analyses for *S. aureus*. In *S. aureus*, we identified five acetylated and 108 propionylated proteins that were significantly and uniquely enriched (Figure 7A, Tables S12 and S13) from the 2864 proteins in the proteome.^51^ Notably, we did not observe any overlap between acetylated and propionylated proteins in *S. aureus*. We speculate on possible reasons for the limited number of acetylated proteins in the Discussion. We performed KEGG pathway enriched analysis for both acylomes as described in Materials and Methods. Acetylated proteins were enriched in biotin metabolism, fatty acid biosynthesis and metabolism, and cofactor biosynthesis, suggesting that acetylation in *S. aureus* regulates processes that are important for growth (Figure 7B, Table S14). Propionylated proteins were enriched in pathways for pyruvate metabolism, glycolysis/gluconeogenesis, various amino acid metabolism and degradation pathways, fatty acid degradation, and others, demonstrating a clear role in central metabolism (Figure 7B, Table S15). Overall, acylated proteins were distributed across diverse functional categories, demonstrating that lysine acylation broadly regulates cellular processes in *S. aureus*. To compare our MRSA acetylome to a methicillin-sensitive *S. aureus* (MSSA) strain, we directly compared our dataset to a published MSSA acetylome.^33^ We identified 27 proteins that were uniquely acetylated in the MRSA strain (Figure 7C), which were enriched for KEGG pathways involved in fatty acid biosynthesis, nucleotide metabolism, the tricarboxylic acid (TCA) cycle, biotin metabolism, peptidoglycan biosynthesis, and branched-chain amino acid degradation, suggesting that strain-specific acetylation in MRSA preferentially targets metabolic and cell envelope functions (Figure 7D, Table S16). Overall, these findings further illustrate how our method effectively captures biologically relevant PTMs across organisms from both Gram-positive and Gram-negative bacteria.

**Figure 7:**
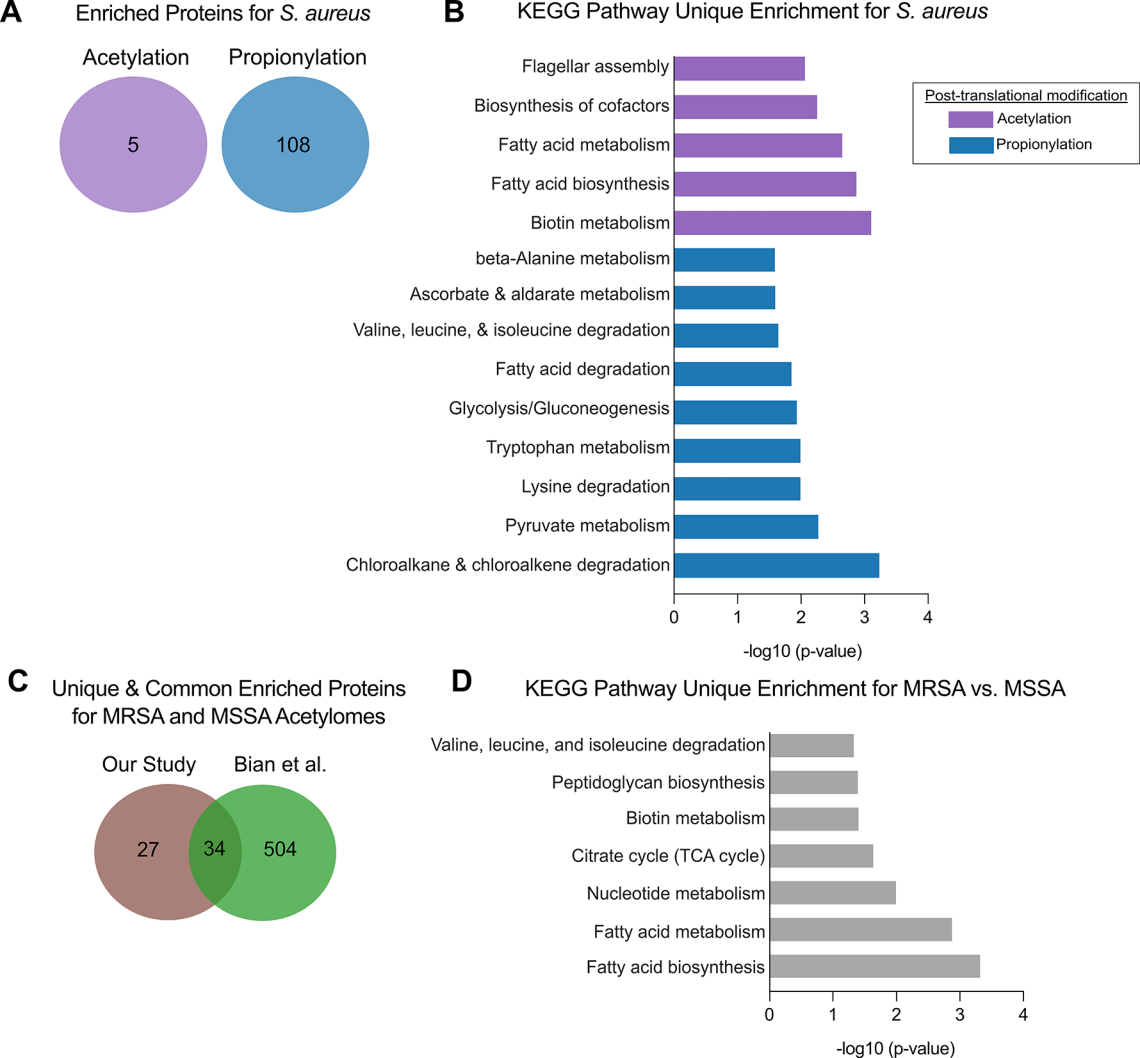
Analysis of unique enriched acetylated and propionylated proteins in *S. aureus*. Proteins significantly enriched for acetylation and propionylation in *S. aureus* were identified and compared to determine unique and common acylated proteins (as described in Materials and Methods). (A) Venn diagram showing enriched acetylated (purple) and propionylated (blue) proteins. (B) KEGG pathway enrichment was performed as described in Materials and Methods. Statistically significant KEGG pathways for acetylated proteins (purple) and propionylated (blue) were determined as having a −log_10_-transformed *p*-value (*x*-axis) of 1.3 and above. (C) Comparative analysis of the MRSA acetylome from this study and the MSSA acetylome reported by Bian et al. The Venn diagram shows the number of acetylated proteins unique to MRSA (27), unique to MSSA (504), and shared between both strains (34). (D) Bar graph depicts KEGG pathways significantly enriched among the 27 MRSA-unique acetylated proteins. Statistically significant KEGG pathways were determined as having a −log_10_-transformed *p*-value (*x*-axis) of 1.3 and above.

## Discussion

In this study, we present the first application of bioorthogonal click chemistry to define specific acylomes in *P. aeruginosa* UCBPP-PA14 and methicillin-resistant *S. aureus* (MRSA). This represents the first report of the propionylome and butyrylome in a *Pseudomonas* strain and the acetylome and propionylome in MRSA. Our method is fast, efficient, and cost-effective, offering high-resolution quantitative analysis while maintaining high specificity for acylated proteins. Previous acylome identification studies have relied on anti-acyl-lysine antibody enrichment, which lacks statistical rigor, requires extensive processing, and incurs high costs. In contrast, by comparing material costs across methods, our labeling method costs approximately $8 per sample, compared to $30–$200 per sample for antibody-based approaches,^58,59^ while capturing a comparable or greater proteome coverage. While our method is a successful tool for identifying acylation targets, it does have some limitations. Because the modified residue remains bound to the DBCO-agarose bead after trypsin digestion, site-specific information about acylation cannot be determined via conventional shotgun proteomic analysis of digested peptides. However, the quantitative and statistically robust nature of our approach makes it well-suited for studying the function of uncharacterized acyltransferases and deacylases. By combining our enrichment workflow with genetic deletion or overexpression of putative acyltransferases or deacylases, changes in the acylation profile can be systematically quantified to identify enzyme-substrate interactions. These top candidate protein targets can then be validated biochemically using purified proteins and established *in vitro* acylation assays.^30^

### Acetylation Dynamics and Biological Functions in *Pseudomonas aeruginosa*

Among the three modifications examined, acetylation was the most abundant in *P. aeruginosa*, consistent with the high intracellular concentration of acetyl-CoA, which is a central metabolic intermediate and the primary acetyl donor for acetyltransferases. Correspondingly, acetylated proteins were enriched for pathways including the TCA cycle, propanoate metabolism, and carbon fixation, supporting the role for acetylation in regulating central metabolism in *P. aeruginosa*. In contrast, propionylation and butyrylation were less abundant, likely reflective of lower intracellular concentrations of propionyl-CoA and butyryl-CoA, which are produced from branched-chain amino acid and odd-chain fatty acid degradation or fermentation.^60,61^ Nevertheless, these modifications are likely dynamic, with levels influenced by the growth phase, environmental stress, or nutrient availability.

In this study, propionylated proteins in *P. aeruginosa* were enriched for pathways annotated under vancomycin resistance, suggesting a potential role in cell wall modification and antibiotic evasion. Butyrylated proteins were enriched for folate and phenazine biosynthesis, both critical for secondary metabolism and infection-related physiology.^55,62^ Phenazines are redox-active metabolites that contribute to biofilm maintenance, redox balance, and virulence by generating reactive oxygen species (ROS) that can damage the host cells.^63,64^ We identified butyrylation on two key phenazine biosynthetic enzymes, PhzB2 and PhzM. Because PhzM interacts with PhzS to catalyze the conversion of phenazine carboxylate (PCA) into 5-Me-PCA and then into pyocyanin (PYO),^65^ we hypothesize butyrylation of PhzM may stabilize the PhzM-PhzS complex or modulate its activity, potentially optimizing PYO production. Likewise, butyrylation of PhzB2 may influence flux through the phenazine biosynthetic pathway.^66^ These findings suggest that posttranslational acylation could fine-tune phenazine biosynthesis and, consequently, virulence in *P. aeruginosa*.

As this study provides the first report of the propionylome and butyrylome in *P. aeruginosa*, further investigation is needed to better understand the dynamics these events play in cellular regulation. The substantial overlap among acetylated, propionylated, and butyrylated proteins in *P. aeruginosa* also raises questions about acyltransferase enzyme specificity and potential PTM crosstalk. The observed overlap may indicate that multiple acyl modifications collectively regulate the metabolic state and regulatory responses. It remains to be determined whether the overlap of these modifications results from enzyme promiscuity or from nonenzymatic acylation driven by reactive acyl-CoA thioesters.^30,67^

### Distinct Acylation Patterns in Methicillin-Resistant *Staphylococcus aureus*

Overall, we observed fewer modified proteins in *S. aureus* compared to *P. aeruginosa*. This difference could be due to several factors, including intrinsic biological variability, lower abundance of certain acyl donors, and technical challenges associated with extracting proteins tightly associated with the Gram-positive cell wall.^68^ Additionally, the hydrophobic and cationic nature of many *S. aureus* proteins may increase nonspecific binding to the DBCO moiety of the agarose beads, elevating background signal and complicating our background subtraction process.^68^ In *S. aureus* acetylation primarily influenced biotin and fatty acid biosynthesis, both of which are essential for maintaining energy homeostasis during infection.^69–71^ We also observed acetylated proteins enriched for biosynthesis of cofactor pathways, one such protein being glutamate-1-semialdehyde 2,1-aminomutase, an enzyme required for 5-aminolevulinate production and heme biosynthesis.^72^ Heme is essential for cellular respiration and oxidative stress defenses, but is toxic in excess.^73^ Because eukaryotes use distinct heme biosynthetic enzymes, this pathway in *S. aureus* may represent a potential antibiotic target for controlling MRSA infections.^74^

In contrast, propionylation was more abundant than acetylation in *S. aureus*, with propionylated proteins primarily enriched in pathways related to glycolysis, gluconeogenesis, pyruvate metabolism, and amino acid degradation, suggesting a key role in metabolic regulation analogous to that of acetylation in *P. aeruginosa*. Propionylated proteins were also enriched in pathways for chloroalkane and chloroalkene degradation, which reflect stress response or detoxification mechanisms. We also observed the enrichment of propionylated proteins in ascorbate and aldarate degradation pathways, processes not yet experimentally characterized in *S. aureus*. This highlights the limitations of incomplete genome annotation and the need for careful interpretation of KEGG-based enrichment results. Together, our data suggest that in *S. aureus*, propionylation plays a key role in regulating central metabolism, while acetylation regulates growth, motility, and stress responses. We observed little to no butyrylation in *S. aureus*, likely reflective of the growth inhibitory properties of butyrate on *S. aureus*, along with the lack of enzymes in *S. aureus* required to convert butyrate to its CoA thioester.^51,75^

### Uncharacterized Proteins and Future Directions

A notable and significant portion of acylated proteins identified in both species were annotated as hypothetical. In *P. aeruginosa*, hypothetical proteins accounted for 25.8% of the acetylome, 36.4% of the propionylome, and 38.9% of the butyrylome. Similarly, in *S. aureus*, hypothetical proteins made up 20% of the acetylome and 15.7% of the propionylome. These findings suggest that a vast number of uncharacterized proteins are regulated by acylation. Future studies aimed at defining the functions of these proteins will not only advance our understanding of bacterial regulation via acylation but also uncover novel aspects of bacterial pathogenesis. It would also be valuable to explore how infection-relevant stressors, such as ROS, antibiotics, pH, and decreased oxygen levels, impact acylation dynamics in *P. aeruginosa* and *S. aureus*. While this study analyzed acylomes in planktonic cultures, extending this analysis to host-associated lifestyles, including biofilms, would provide a more complete understanding of how acylation contributes to bacterial adaptation and physiology under host-mimicking conditions.

## Conclusion

In conclusion, we developed a fast, cost-effective, and highly specific bioorthogonal click chemistry method to globally profile lysine acylation in bacterial strains of *P. aeruginosa* and methicillin-resistant *S. aureus* (MRSA). This approach enabled, for the first time, characterization of the propionylome and butyrylome in *P. aeruginosa* UCBPP-PA14 as well as the acetylome and propionylome in an MRSA strain. Enrichment analysis revealed distinct patterns of acylation between these two pathogens, highlighting unique roles for acylation in metabolic regulation, virulence, antibiotic resistance, and adaptation to host and environmental stressors. Our method provides quantitative fold-change measurements, expands the coverage of the acylome compared to antibody-based enrichments, and allows for the discovery of underexplored regulatory networks. As acylation emerges as a critical regulator of bacterial metabolism and physiology, it is our hope that others will utilize this method to aid in new discoveries of bacterial acylation and define its role in pathogenesis, antibiotic resistance, and novel therapeutic target identification.

## Supplementary Material



































## Notes

Table S1: Total acetylated proteins identified in *Pseudomonas aeruginosa* (XLSX)
Table S2: Total propionylated proteins identified in *P. aeruginosa* (XLSX)
Table S3: Total butyrylated proteins identified in *P. aeruginosa* (XLSX)
Table S4: Unique acetylated proteins in *P. aeruginosa* compared to propionylated and butyrylated proteins (XLSX)
Table S5: Unique propionylated proteins in *P. aeruginosa* compared to acetylated and butyrylated proteins (XLSX)
Table S6: Unique butyrylated proteins in *P. aeruginosa* compared to acetylated and propionylated proteins (XLSX)
Table S7: KEGG pathway enrichment of unique acetylated proteins from Table S5 in *P. aeruginosa* (XLSX)
Table S8: KEGG pathway enrichment of unique propionylated proteins from Table S6 in *P. aeruginosa* (XLSX)
Table S9: KEGG pathway enrichment of unique butyrylated proteins from Table S7 in *P. aeruginosa* (XLSX)
Table S10: Comparison of *P. aeruginosa* acetylome with previously published datasets (XLSX)
Table S11: KEGG pathway enrichment of *P. aeruginosa* acetylated proteins from this study in common with published datasets (XLSX)
Table S12: Total and unique acetylated proteins in *S. aureus* (XLSX)
Table S13: Total and unique propionylated proteins in *S. aureus* (XLSX)
Table S14: KEGG pathway enrichment of unique acetylated proteins in *S. aureus* (XLSX)
Table S15: KEGG pathway enrichment of unique propionylated proteins in *S. aureus* (XLSX)
Table S16: KEGG pathway enrichment of acetylated proteins unique to or shared between MRSA and MSSA strains (XLSX)
Figure S1–S7: Volcano plots showing background-subtracted mass spectrometry data for acetylomes, propionylomes, and butyrylomes of *P. aeruginosa* and *S. aureus*, structures of chemical probes, and supplemental protein gels (PDF)
