# Supplementary material for: Bioorthogonal Click Chemistry for Antibody-Free Profiling of Acetylation, Propionylation, and Butyrylation in Pseudomonas aeruginosa and Methicillin-Resistant Staphylococcus aureus
Source: ACS Infect Dis. 2026 Feb 16;12(3):1142–54. doi: 10.1021/acsinfecdis.5c00985 (PMC12993845; doi:10.1021/acsinfecdis.5c00985)
Supplement: Supplementary file 17 [file id5c00985_si_017.pdf]

SUPPLEMENTAL INFORMATION FOR:

Bioorthogonal Click Chemistry for Antibody-Free Profiling of Acetylation, Propionylation, and Butyrylation in *Pseudomonas aeruginosa* and Methicillin-Resistant *Staphylococcus aureus.*

Haley N. Monacchio[a†], Ritika S. Shah[b†], Christian F. Montes[c], Grace Z. Wang[d], Justin W. Walley[c], Chelsey M. VanDrisse[b*]

[1]University of Georgia, Department of Microbiology, Athens, GA USA
[2]University of Georgia, Department of Genetics, Athens, GA USA
[3]Iowa State University, Plant Pathology, Entomology and Microbiology, Ames, IA USA
[4]California Institute of Technology, Division of Chemistry and Chemical Engineering, Pasadena, CA, USA

*Correspondence: Chelsey M. VanDrisse, email: cmvd@uga.edu, Department of Genetics, University of Georgia, Davison Life Sciences, 120 E Green Street, Athens, GA 30602 USA
[†]Authors contributed equally

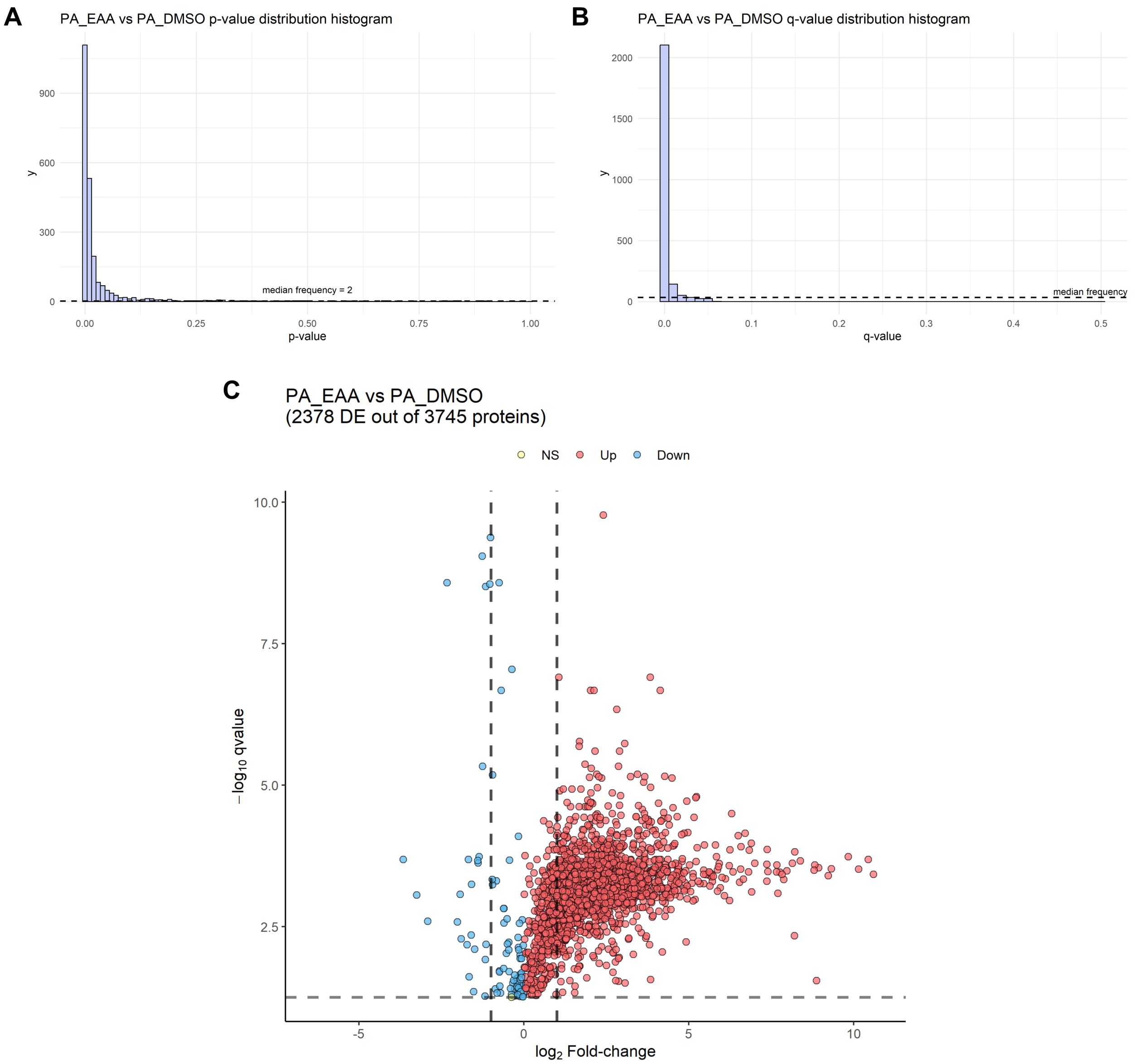

Figure S1. Statistical validation and differential enrichment analysis of acetylated proteins in *P. aeruginosa* using ethyl azidoacetate (EAA) labeling. (A) Histogram showing the distribution of p-values for differential protein abundance between ethyl azidoacetate (EAA)-enriched samples and DMSO vehicle controls in *P. aeruginosa*. (B) Histogram of q-value (FDR-adjusted p-value) distribution for the same protein comparison. (C) Volcano plot illustrating differential enrichment of proteins. Each point represents an individual protein. Proteins significantly enriched over DMSO (above background) are shown in red, those significantly below in blue, and non-significant proteins in yellow. Dashed lines indicate thresholds for significance and fold-change.

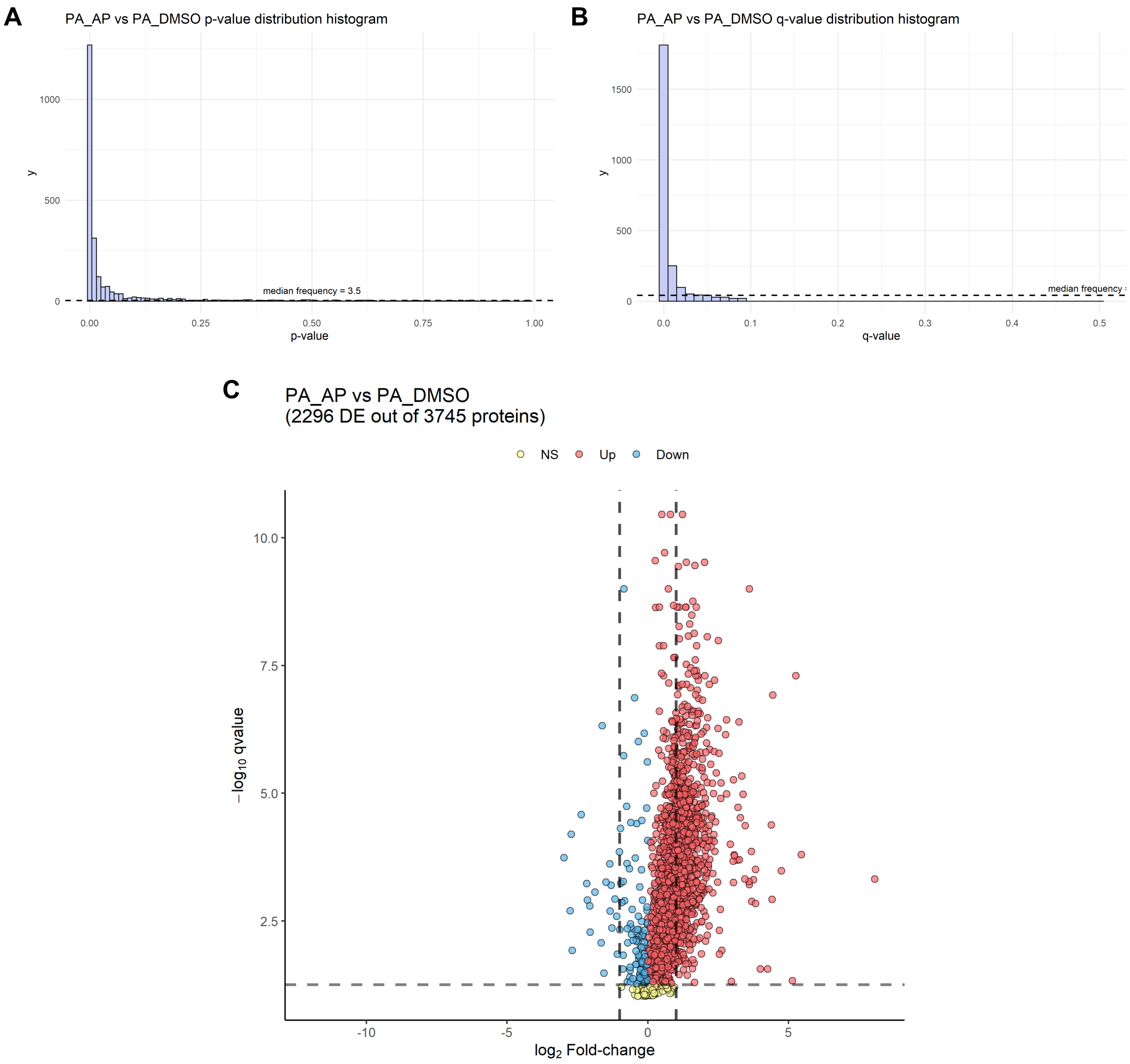

Figure S2. Statistical validation and differential enrichment analysis of acetylated proteins in *P. aeruginosa* using azidopropanoic acid (AP) labeling. (A) Histogram showing the distribution of p-values for differential protein abundance between azidopropanoic acid (AP)-enriched samples and DMSO vehicle controls in *P. aeruginosa*. (B) Histogram of q-value (FDR-adjusted p-value) distribution for the same protein comparison. (C) Volcano plot illustrating differential enrichment of proteins. Each point represents an individual protein. Proteins significantly enriched over DMSO (above background) are shown in red, those significantly below in blue, and non-significant proteins in yellow. Dashed lines indicate thresholds for significance and fold-change.

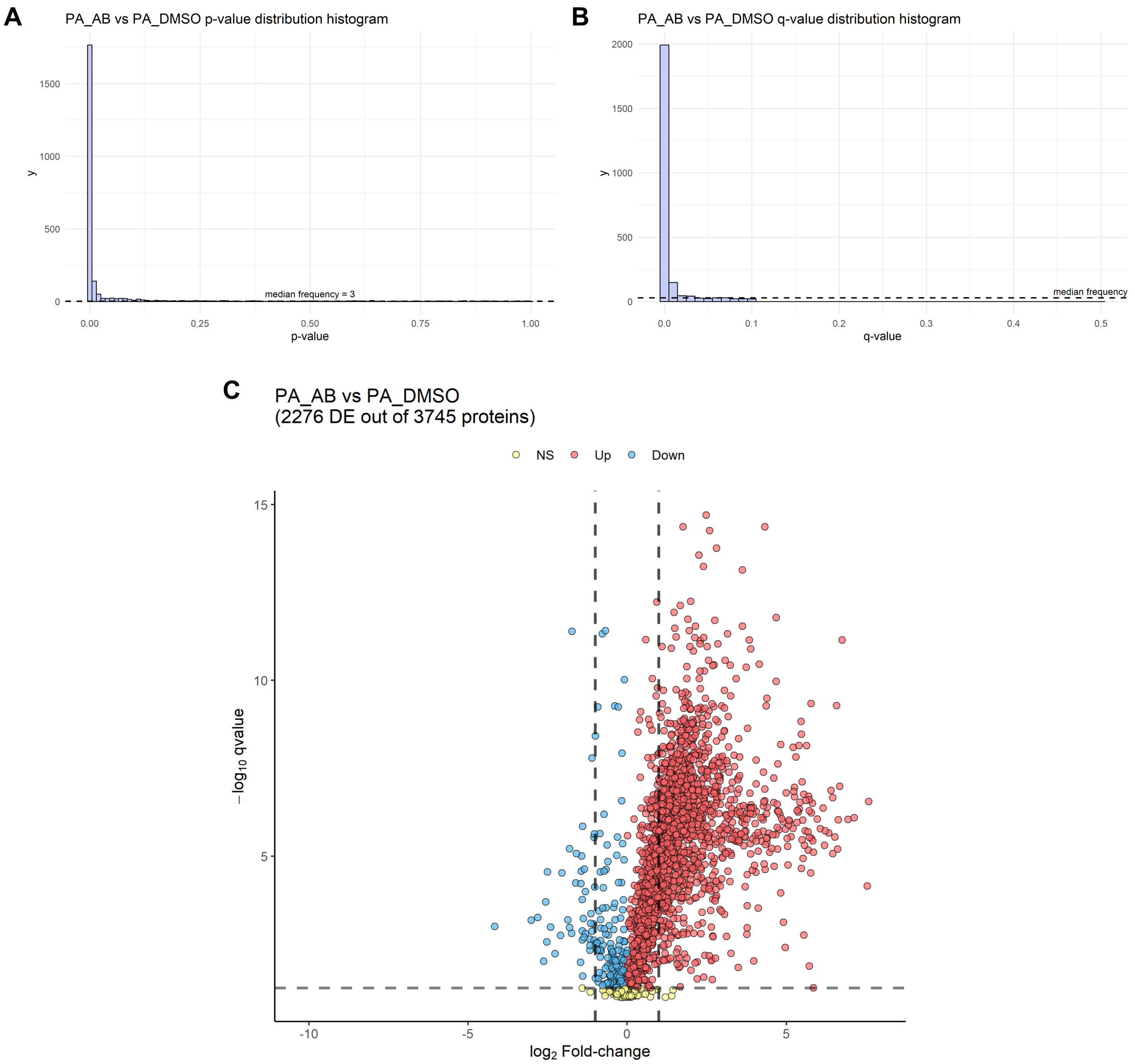

Figure S3. Statistical validation and differential enrichment analysis of acetylated proteins in *P. aeruginosa* using azidobutyric acid (AB) labeling. (A) Histogram showing the distribution of p-values for differential protein abundance between azidobutyric acid (AB) -enriched samples and DMSO vehicle controls in *P. aeruginosa*. (B) Histogram of q-value (FDR-adjusted p-value) distribution for the same protein comparison. (C) Volcano plot illustrating differential enrichment of proteins. Each point represents an individual protein. Proteins significantly enriched over DMSO (above background) are shown in red, those significantly below in blue, and non-significant proteins in yellow. Dashed lines indicate thresholds for significance and fold-change.

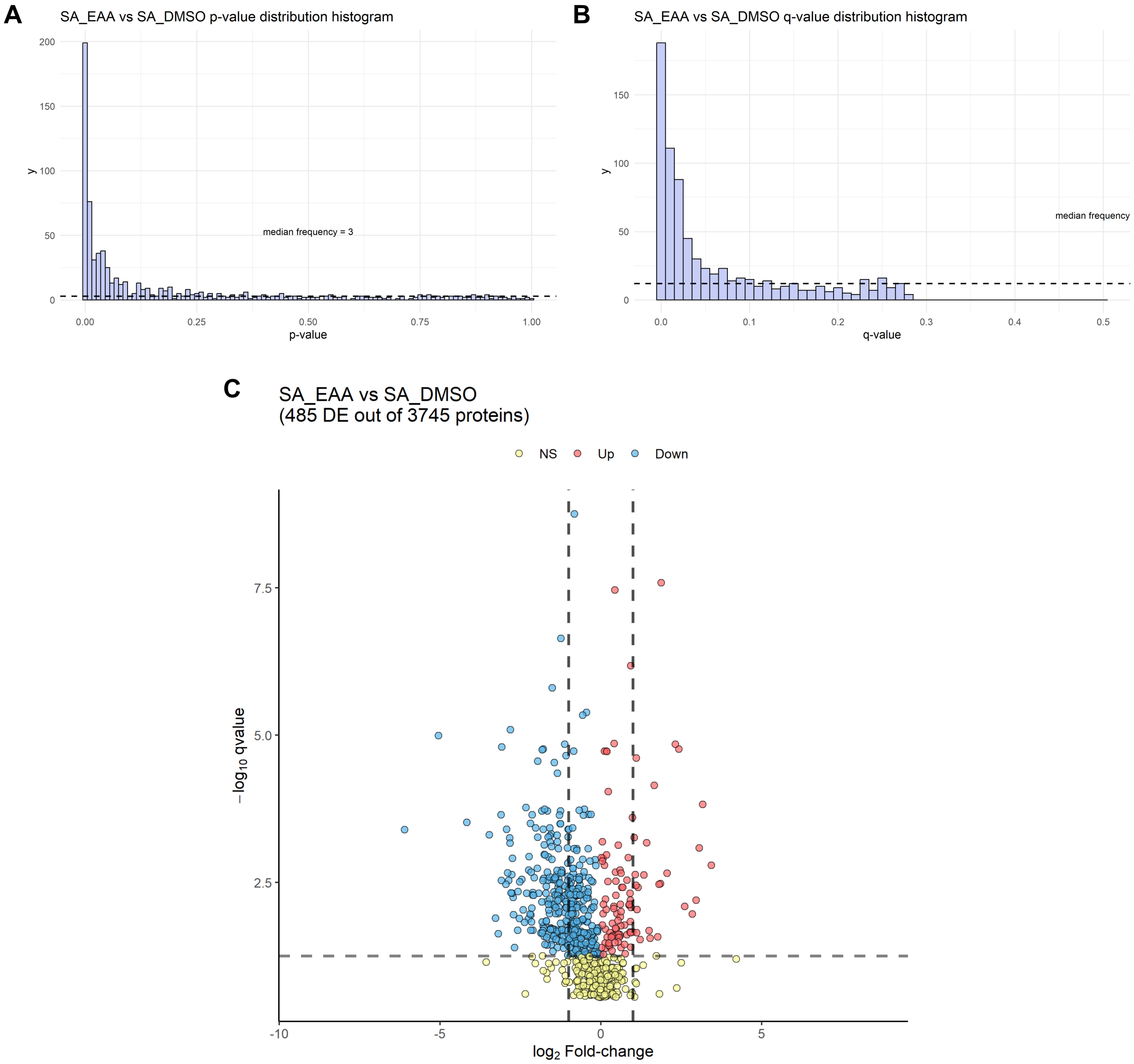

Figure S4. Statistical validation and differential enrichment analysis of acetylated proteins in *S. aureus* using ethyl azidoacetate (EAA) labeling. (A) Histogram showing the distribution of p-values for differential protein abundance between ethyl azidoacetate (EAA)-enriched samples and DMSO vehicle controls in *S. aureus*. (B) Histogram of q-value (FDR-adjusted p-value) distribution for the same protein comparison. (C) Volcano plot illustrating differential enrichment of proteins. Each point represents an individual protein. Proteins significantly enriched over DMSO (above background) are shown in red, those significantly below in blue, and non-significant proteins in yellow. Dashed lines indicate thresholds for significance and fold-change.

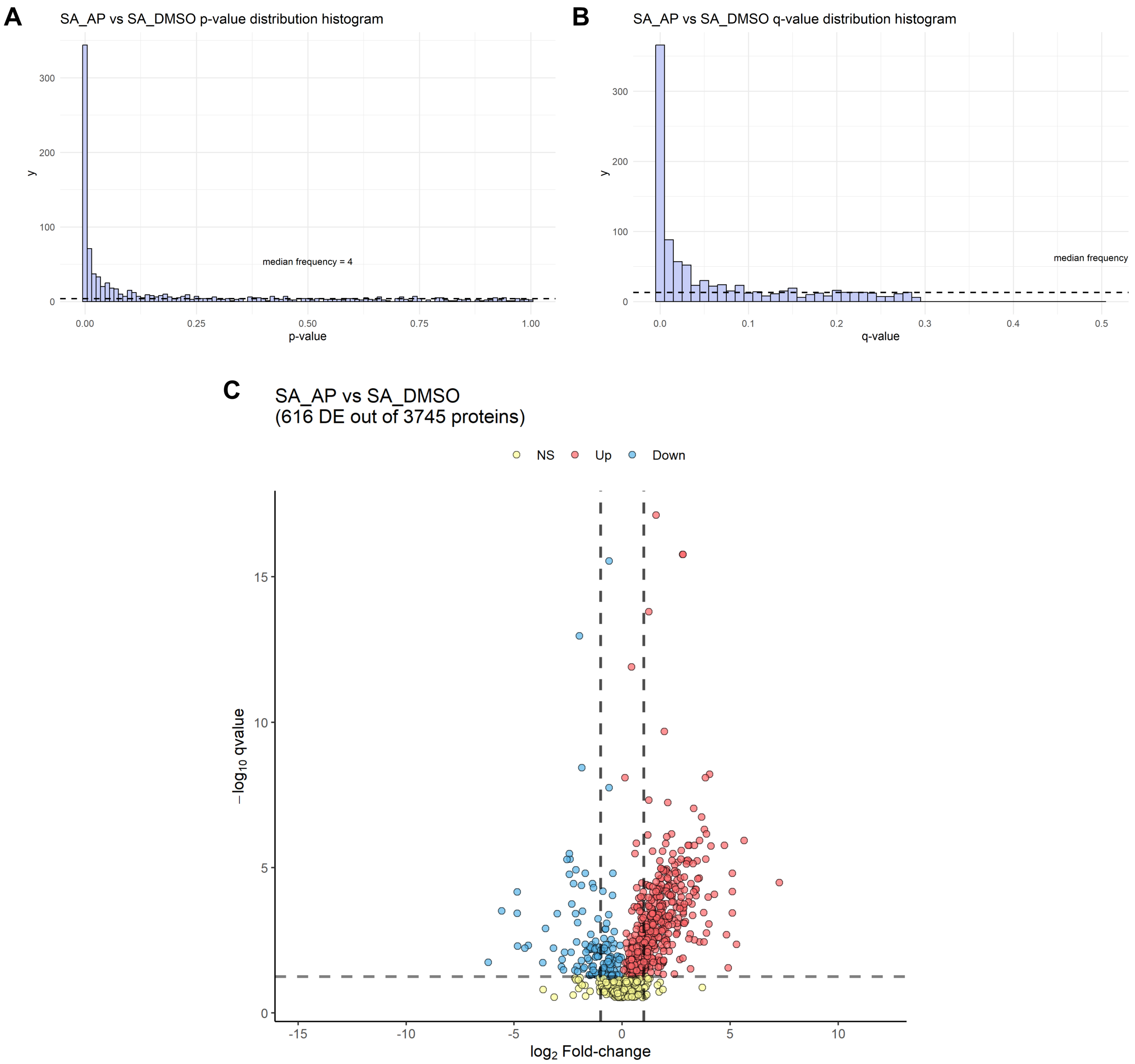

Figure S5. Statistical validation and differential enrichment analysis of acetylated proteins in *S. aureus* using azidopropanoic acid (AP) labeling. (A) Histogram showing the distribution of p-values for differential protein abundance between azidopropanoic acid (AP)-enriched samples and DMSO vehicle controls in *S. aureus*. (B) Histogram of q-value (FDR-adjusted p-value) distribution for the same protein comparison. (C) Volcano plot illustrating differential enrichment of proteins. Each point represents an individual protein. Proteins significantly enriched over DMSO (above background) are shown in red, those significantly below in blue, and non-significant proteins in yellow. Dashed lines indicate thresholds for significance and fold-change.

C2-probes

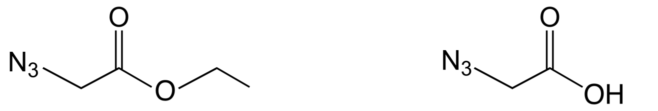

ethyl azidoacetate azidoacetic acid

C3-probes

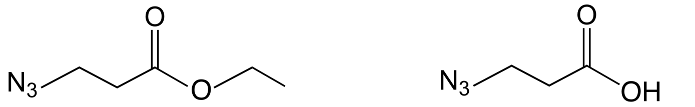

ethyl azidopropionate azidopropionic acid

C4-probes

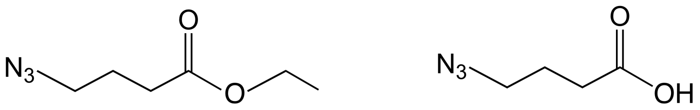

ethyl azidobutyrate azidobutyric acid

Figure S6. Chemical structures of azidoacyl probes used for click chemistry labeling in this study. Each probe was utilized in both the ester (ethyl azidoacetate, ethyl azidopropionate, and ethyl azidobutyrate) and corresponding acid (azidoacetic acid, azidopropionic acid, and azidobutyric acid) forms. The varying acyl chain lengths allow comparison of acetyl-, propionyl-, and butyryl- labeling of bacterial proteins to assess acylomes profiles.

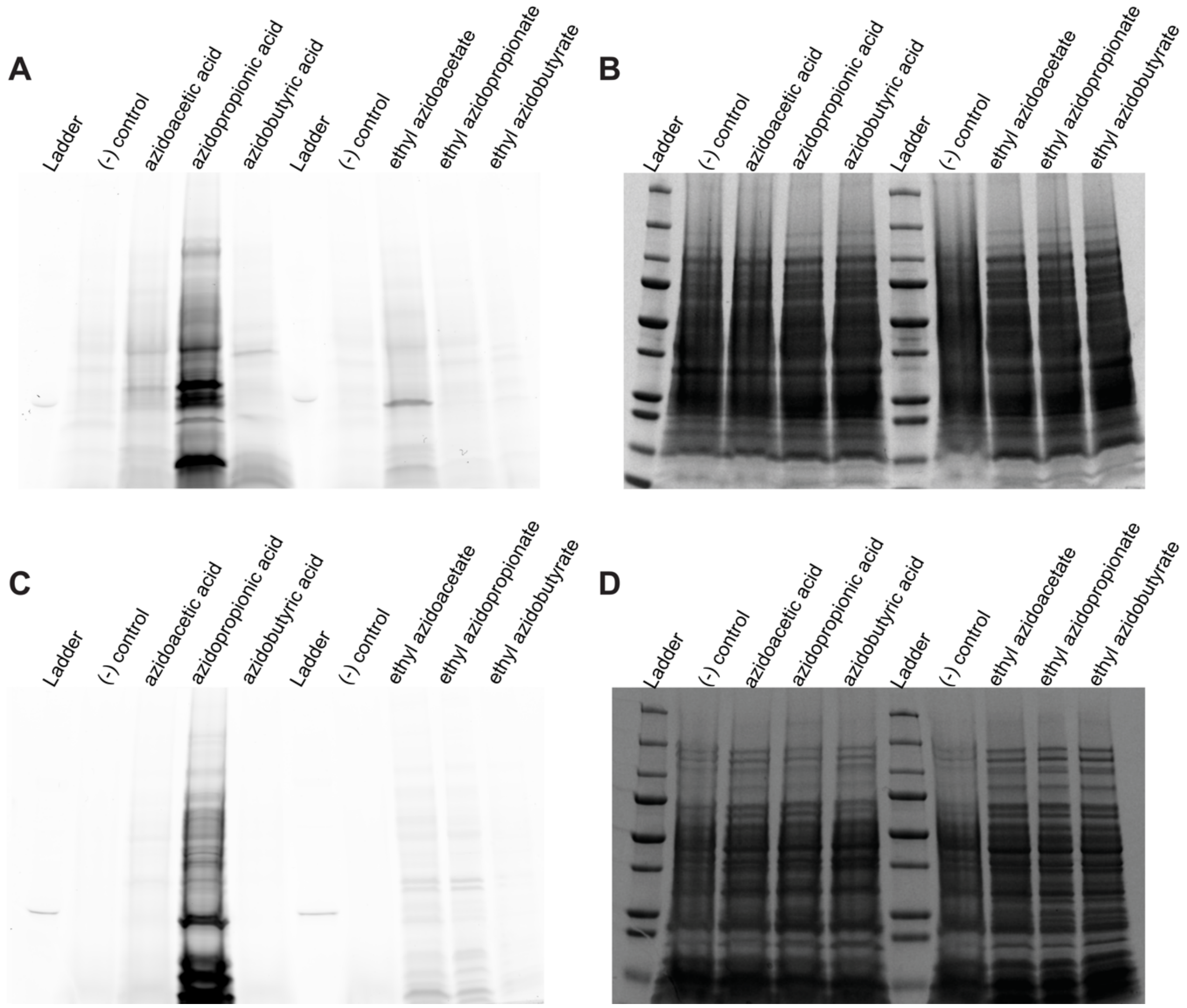

Figure S7. Visualization of protein acetylation, propionylation, and butyrylation in *P. aeruginosa* and *S. aureus* normalized to azidopropanoic acid samples. *P. aeruginosa* **(A & B)** and *S. aureus* **(C & D)** were treated with azidoacids and ethylazidoacids for 30 minutes and samples were collected (as described in Materials and Methods). The acetylated, propionylated, and butyrylated proteins were conjugated to TAMRA alkyne fluorophore via click chemistry and the gels were visualized using the Typhoon **(A & C)**. The same gels were then stained and destained with Coomassie Blue and Fairbanks Destaining Solution, respectively; the stained gels were then imaged **(B & D)**. Gels are identical to images in Figure 4 of main text, but are normalized brightness and contrast intensities to the azidopropanoic acid samples.
